# Funerary vs. domestic vessels from the Hallstatt period. A study on ceramic vases from the Milejowice settlement and the Domasław cemetery

**DOI:** 10.1038/s41598-024-70219-7

**Published:** 2024-08-27

**Authors:** Angelina Rosiak, Anna Józefowska, Joanna Sekulska-Nalewajko, Jarosław Gocławski, Joanna Kałużna-Czaplińska

**Affiliations:** 1 Institute of General and Ecological Chemistry, Faculty of Chemistry, Lodz University of Technology, Żeromskiego 116, 90-924 Lodz, Poland; 2 Institute of Archaeology and Ethnology, Polish Academy of Sciences, Al. Solidarności 105, 00-140 Warszawa, Poland; 3 Institute of Applied Computer Science, Faculty of Electrical, Electronic, Computer and Control Engineering, Lodz University of Technology, Stefanowskiego 18, 90-537 Lodz, Poland

**Keywords:** Hallstatt ceramics, Funerary vs. domestic vessels, Chromatographic analyses, Statistical analyses, Lipids, Analytical chemistry

## Abstract

Clay vessels have a wide variety of functions in social activities in the Hallstatt period. In addition to food storage and processing, they were used for ritual purposes and as funerary vessels. The paper presents the results of archaeological and chromatographic studies of 31 vases from two different Hallstatt culture sites in lower Silesia (Poland). The investigations included vessels fragments from the Domasław cemetery and from the Milejowice settlement. The chromatographic analyses focused on fatty acids and biomarkers and made it possible to identify the most likely sources of substances they came into contact with during use. The c-means and hierarchical cluster analyses showed that grave vessels differed from settlement ceramics. Thus, conclusions on the diverse vessel functions could be made.

## Introduction

Interpreting the functions and uses of prehistoric ceramic containers is largely intuitive^1^. The particular shapes and technological properties make certain pottery types suitable for specific activities related to drinking, serving, storing, and preparing food and/or beverages. Morphologically, pottery might be divided into vases, pots, bowls, cups and other vessel types. However, the functions this classification implies may differ from their actual use and they may contain completely different products in particular contexts.

The south-western Poland’s Hallstatt period (ca. 8–6th BC) saw deep social transformations, i.e., the emergence of local “elites”—probably due to trade development and intense external contacts. The import influx, the gradual adoption of cultural ideas from the east and the south, and, above all, the population’s growing wealth resulted in lifestyle changes. This transformation might be traced in settlement evidence through new forms of status display and in cemeteries, where the practices of the Hallstatt elites were imitated. The emergence of new pottery types, such as vases with conical rims and funnel-shaped necks, plate-like bowls, and *rhyta*, was associated with these new patterns and social “needs” during feasting or ceremonies. Unfortunately, neither the daily-use nor offering vessels provides reliable information on their contents or usage patterns. Therefore, by comparing the settlement and funerary contexts of pottery using chromatographic analyses, we sought to identify the functions of these containers and, thus, better understand the ancient practices.

For this purpose, two key sites of the Hallstatt period, located close to each other were selected: a settlement in Milejowice, site 19, partly fenced with palisade-like circular structures and a cemetery in Domasław, sites 10/11/12, with richly furnished chamber graves. These Silesian sites were excavated in 1999–2003 (Milejowice) and 2006–2008 (Domasław) by the Institute of Archeology and Ethnology of the Polish Academy of Sciences in Wrocław.

Buildings at the extensive Milejowice settlement were organised in clusters, with a separated zone surrounded by a regular circular palisade. This part cannot be interpreted as fortifications but rather as the elite’s seat, inhabited by a privileged group displaying their high social and economic status. The site consisted of numerous residential and non-residential ground-level buildings and dug-in structures, such as storage pits, wells, a metallurgy workshop, votive pits and resource pits. Pottery, including the “luxurious” types with painted or graphite-coated surfaces, might also have been manufactured here^2–6^.

At the Hallstatt cemetery in Domasław, ca. 800 cremation burials were discovered, including 300 chamber graves with impressively rich personal ornaments, weapons, toiletry sets, tools, bronze vessels, and a large amount of luxurious pottery. The graves contained selected vessel types—for food, drinks and other offerings. They were often exceptionally decorative—painted or with glossy, graphite-coated surfaces, imitating metallic vessels—deposited in a predefined position and number^7,8^.

The authors intended to compare the use of vase-shaped vessels in funerary and settlement contexts and from features interpreted as residential, votive or connected to household/production. The main goal was to determine whether it is possible to distinguish between funerary and household pottery considering the products it contained and whether any significant differences indicate the function of these storage vessels in different contexts.

## Materials

The paper focuses on ceramic vase-like vessels found at the archaeological sites from the Hallstatt period: the Milejowice settlement and the Domasław cemetery. The analyses included 31 vases (16 funerary and 15 settlement finds). The conclusions, however, were based on the results obtained from 120 vessels of various types from these two sites examined as part of the project. Table 1 shows the characteristics of the studied vases.

**Table 1:** Characteristics of the studied vases.

Sample number	Object number	Vessel number	Comments
Domasław cemetery			
3	360	5	a vase with funnel-shaped rim and conical neck, graphite-coated, decorated; a bronze dipper, bowl and eight cups inside, a plate-shaped bowl on the vase
4	384	13	a vase with funnel-shaped rim and conical neck, graphite-coated, decorated; a cup and a plate-shaped bowl inside, two cups under
10	8893	10	a vase with funnel-shaped rim and conical neck, decorated
12	8919	7	a vase with funnel-shaped rim and conical neck, graphite-coated, decorated; a bronze vessel inside
90	1300	3	a vase with a funnel-shaped rim and conical neck
91	390	7	a vase with funnel-shaped rim and conical neck, graphite-coated, decorated
93	4270	22	a vase with funnel-shaped rim and conical neck, graphite-coated, decorated
94	2849	4	a vase with funnel-shaped rim and conical neck, graphite-coated, decorated
95	6366	5	a vase with funnel-shaped rim and conical neck, graphite-coated, decorated; a bowl inside
101	7429	6	a vase with a funnel-shaped rim and conical neck, graphite-coated, decorated
104	7429	20	a vase with funnel-shaped rim and conical neck, graphite-coated, decorated; four cups inside
105	7429	25	a vase with a conical neck and slightly everted rim, graphite-coated, decorated; covered with a bowl
106	384	14	a vase with a conical neck and slightly everted rim, graphite-coated, decorated; covered with a lid
108	4270	4	a vase with a conical neck and straight rim, graphite-coated, decorated
111	8905	9	a vase with funnel-shaped rim and conical neck, graphite-coated, decorated; two cups and a bowl underneath
113	4384	7	a vase with funnel-shaped rim and conical neck, graphite-coated, decorated; two cups inside
Milejowice settlement			
39	53/N	1	a bulbous vase with handles
40	53/N	2	a bulbous vase with handles
42	65/N	1	a vase with a funnel-shaped rim and conical neck, decorated; with vessels, animal bones, an axe fragment, a bronze artifact and raw material inside
43	442/S	1	a vase with a short, funnel-shaped rim and conical neck; with other vessels and animal bones
46	1642/S	2	a vessel with a funnel-shaped rim and conical neck, graphite-coated, decorated
47	751/S	1	a vase with a sigmoid rim, with a handle, graphite-coated, decorated
49	751/S	3	a vase with a funnel-shaped rim and conical neck, painted, secondarily burnt
53	585/N	1	a vase with a funnel-shaped rim and conical neck, decorated
55	585/N	3	a vase with a funnel-shaped rim and conical neck, decorated
58	1493/S	1	a vase with a funnel-shaped rim and conical neck, graphite-coated, decorated
61	264/S	3	a vase with a funnel-shaped rim and conical neck, graphite-coated, decorated
70	367a/S	1	an s-shaped vase, decorated
72	588/N	1	a vase with a funnel-shaped neck, with a handle
79	301/S	2	a vase with a funnel-shaped rim and conical neck
86	204/S	1	a vase with a funnel-shaped rim and conical neck, painted, decorated, secondarily burnt

**Figure 1:**
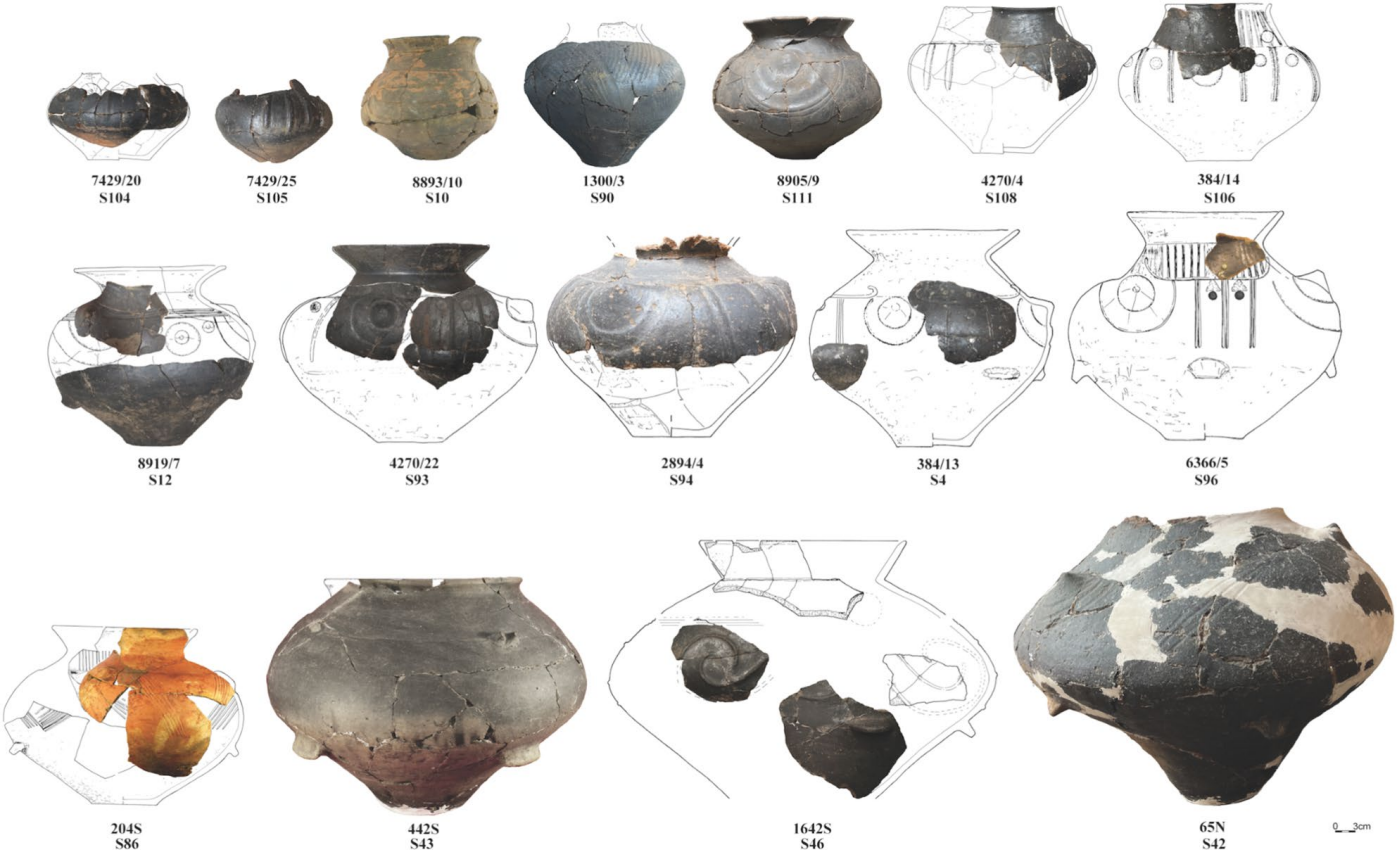
Selected vases from the Domasław cemetery and the Milejowice settlement (S-sample).

The vases (Fig. 1) are medium and large vessels, with rounded or close to biconical bodies and varying neck shapes. Most of them had funnel-shaped rims and cone-shaped necks. Another type featured conical necks and straight or slightly everted rims, while others had more S-shaped profiles and sometimes handles.

Their diameters ranged to over 45 cm at the cemetery and up to 60 cm at the settlement. Vessels with funnel-shaped rims constituted the numerous and characteristic group of vessels. They frequently occurred in Silesia during the Hallstatt period, and their surface treatment and decorations indicated an influence from the Hallstatt zones^9^. Almost all cemetery vases and some from the settlement (46, 47, 58, 61) have graphite-coated surfaces. The upper parts of bodies are often ornamented with slanting, vertical or horizontal grooves or incisions, less often with dimples. Pushed-out or attached conical knobs surrounded by semicircular grooves also occurred, as well as grips, handles, and ribs. On vessel 46, they formed spectacular spiral arrangements. Some vessels are painted. Within the palisade-surrounded area (43, 46, 47, 49), painted ware and vases with funnel-shaped rims and cone-shaped necks were 2–4 times more numerous than in the other settlement clusters^10^.

At the settlement, the studied vases occurred in features interpreted as votive (42, 43, 46), sunken structures of residential or economic character (47, 49, 53, 55, 58, 70, 72, 79, 86), a well (39, 40), a bronze-working workshop (61), and a storage pit (70). In graves, vases were deposited in precisely defined contexts. All occurred in grave chambers except for vessel 90, which was placed outside the chest. Vases with funnel-shaped rims were parts of sets consisting of two vessels of this type located in grave centres. Those with cone-shaped necks were found in the eastern parts (105, 106, 108). Both were part of drinking sets placed in the graves. Vases used as urns were not included in the analysis. They contain a much wider range of compounds, including those of an intoxicating nature.

## Methods

### Chromatographic analyses

A gas chromatograph (6890N GC System, Agilent Technologies) coupled to a mass spectrometer (MS 5973 Network Mass Selective Detector, Agilent Technologies) was used to identify organic compounds in vessel samples.

Samples collected from fragments of ceramic vessels were pre ground and then ground to powder in a mortar. The weighted ground material (5 g) was extracted in a Soxhlet apparatus for 4 h. The extraction mixture contained two solvents: methylene chloride and methanol (200 mL, 2:1 v/v) and 100 μL of standard solution (tetracosane, 1 mg/mL). The extracted lipid fraction was evaporated to dryness and then dissolved in 2 mL of hexane. Portions of 0.5 mL extract were transferred to chromatography vials and evaporated from atmospheric pressure in a stream of nitrogen to dryness. The obtained dry residue was subjected to derivatisation (silylation). 100 μL of a derivatising mixture composed of two reagents was used to convert the analytes into their volatile derivatives (TMS esters), and the reagents used were N,O-bis(trimethylsilyl)trifluoroacetamide and trimethylchlorosilane (100:1 v/v). The sample with the mixture was heated for 30 min at 75 °C. Following this process, 300 μL of hexane was added to the sample and GC-MS analysis was performed.

The analytes were separated on an HP-5MS (5%-diphenyl-95%-dimethylpolysiloxane) column at a carrier gas (He) flow rate of 0.9 mL/min. The injected sample volume was 1 μL. The injector was operated in a mode without separating the carrier gas flow. The gas chromatograph oven was programmed as follows: the initial temperature was 60 °C, with a temperature increase of 12 °C/min until a final temperature of 300 °C was reached. The parameters of the mass spectrometer were as follows: the temperatures of the ion source and mass analyser were 230 °C and 150 °C, respectively. Mass spectra were acquired in electron ionisation (EI) mode at a potential of 70 eV and a sweep range of 50–550 m/z.

Qualitative analysis of organic acids and biomarkers was performed using Wiley and NIST08 mass spectral libraries and commercially available standards. Quantitative analysis of fatty acids was performed using the internal normalisation method.

### Numerical analysis of fatty acid and biomarker data

A set of fatty acids and fatty acid proportions from components indicated by the Student’s t test method was selected to differentiate between the chemical composition of vase samples from the settlement and the cemetery areas. Furthermore, in this study, these selected components were named feature subset 1. The second, alternative feature subset (subset 2) of acid-related features was selected according to the criterion of variable importance in projection (VIP) determined by the supervised partial least squares—discriminant analysis (PLS-DA) modelling method. A VIP score greater than 1 is a typical rule for selecting relevant variables^11^ and might be considered a significant contribution to the explained variable. Scaling and normalisation procedures were used for data pretreatment.

Unsupervised data classification in two clusters has been applied in the feature space of the selected VIP components. The fuzzy *C*-means (FCM) method^12,13^ was used to perform classification, allowing the assessment of the probability of an archaeological sample belonging to both groups (cemetery and settlement).

For clustering quality evaluation, the fuzzy silhouette index (SI), Dunn’s partition coefficient (DC) and partition entropy index (PEI) were used. The SI^14,15^ is calculated using the mean intra-cluster distance (${a}_{j}$) and the mean nearest-cluster distance (${b}_{j}$) for each data sample as shown in Eq. (1).

1 $$SI = \frac{1}{n}\mathop \sum \limits_{j = 1}^{n} \frac{{b_{j} - a_{j} }}{{max\left\{ {a_{j} ,b_{j} } \right\}}} ,$$

where the distances are expressed by logical operations on the sample membership degrees. An SI value equal to 1 indicates perfect cluster separation; values near 0 indicate overlapping clusters.

Similarly, the Dunn coefficient^12^ is the ratio of the smallest distance between any two clusters to the largest intra-cluster distance found within any cluster, as in Eq. (2).

2 $$DC = \frac{{\min \limits_{i<j\leq m}d\left( {C_{i} ,C_{j} } \right) }}{{\max \limits_{x, y}d\left( {x,y} \right) }} ,$$

where m is the number of clusters, ${C}_{i} ,{C}_{j}$ are two compared clusters, d – the Euclidean distance between the clusters or two data samples *x*, *y* in one cluster. When the Dunn index is always above zero, greater values indicate better clustering.

The partition entropy index (PEI)^16,17^ shown in Eq. (3) measures the overlap among clusters.

3 $$PEI = \frac{1}{n} \mathop \sum \limits_{i = 1}^{m} \mathop \sum \limits_{j = 1}^{n} u_{ij} log_{a} \left( {u_{ij} } \right)/m ,$$

where $U={[{u}_{ij}]}_{m\times n}$ represents the fuzzy partition matrix of the *C*-means method with ${u}_{ij}$ as the membership degree of a data sample ${x}_{j}$, and $m$—the number of clusters, logarithm base $a>1$. The range of values for PEI is $[0, 1]$. The closer the PEI value is to 0, the harder and therefore less fuzzy the clustering is.

To verify the consistency of fuzzy C-means clustering prediction with actual division of the examined vessels, the overall accuracy parameter ACC was applied (Eq. 4)

4 $$ACC = \frac{{\mathop \sum \nolimits_{i = 1}^{m} M_{ii} }}{{\mathop \sum \nolimits_{i,j = 1}^{m} M_{ij} }} ,$$

where ${M}_{m\times m}$ is the confusion matrix^18^ in the case of $m$ classes (clusters).

Statistical analyses were performed using the R language directly in the R Studio environment and the MetaboAnalyst software (https://www.metaboanalyst.ca). The Partial Least Squares (PLS) regression was performed using the *plsr* function provided by the *pls* library in *R*. The classification and cross-validation were performed using the corresponding wrapper function offered by the *caret* package. The FCM algorithm was implemented using the *ppclust R* library, and the clustering quality indices were computed using appropriate functions from the *fclust* library.

A permutation test based on prediction accuracy was performed to assess the significance of class discrimination in PLS-DA^19^. In each permutation (100 in total), a PLS-DA model was built between the data (X) and the permuted class labels (Y) using the optimal number of components determined by cross-validation (CV) for the model based on the original class assignment.

Based on binary data on the presence of biomarkers, samples were subjected to clustering analysis using the Ward method and Euclidean distance as a measure of dissimilarity.

## Results

### Chromatographic analyses

The paper focuses on fatty acids and biomarkers that can directly indicate the source of the examined organic residues. The identified fatty acid contents in samples from the Domasław cemetery and the Milejowice settlement are included in Table 2.

**Table 2:** Fatty acids determined in the studied samples (content in %); (-) under the limit of quantification.

Acid symbol	Domasław cemetery																Milejowice settlement														
	3	4	10	12	90	91	93	94	95	101	104	105	106	108	111	113	39	40	42	43	46	47	49	53	55	58	61	70	72	79	86
C6:0	0.32	0.38	1.59	0.21	0.60	0.29	0.64	0.54	0.46	–	0.08	0.10	0.09	0.07	0.14	0.03	0.11	0.14	0.75	0.93	0.29	0.15	0.19	0.35	0.21	0.37	0.27	0.19	0.01	0.17	0.06
C7:0	0.11	0.09	0.42	0.07	0.08	–	0.16	–	0.09	–	–	–	0.04	–	0.10	0.04	–	0.07	0.37	0.36	0.10	0.07	0.10	0.28	0.06	0.20	0.09	0.21	–	0.08	–
C8:0	0.27	0.24	0.78	0.23	0.33	0.22	0.51	0.26	0.26	0.22	0.20	0.10	0.13	0.24	0.26	0.08	0.05	0.22	1.03	0.88	0.28	0.23	0.39	0.41	0.12	0.23	0.29	0.30	0.07	0.20	0.26
C9:0	0.56	0.46	1.09	0.47	0.58	0.32	0.70	0.44	0.39	0.38	0.35	0.28	0.23	0.58	0.33	0.11	0.23	0.54	1.54	1.34	0.68	0.68	0.68	0.50	0.17	0.43	0.50	0.69	0.14	0.33	0.59
C10:0	0.10	0.08	0.27	0.10	0.18	0.19	0.20	0.17	0.14	0.72	0.20	0.32	0.13	0.25	0.18	0.10	0.15	0.26	0.49	0.34	0.22	0.23	0.22	0.14	0.06	0.13	0.23	0.22	0.09	0.16	0.25
C11:0	–	–	–	–	–	–	–	–	–	–	–	–	–	–	–	–	–	–	0.24	0.07	–	–	–	–	–	–	–	–	–	–	–
C12:0	0.49	0.46	0.51	0.37	0.46	0.86	0.57	0.61	0.34	0.58	0.68	0.27	0.41	0.82	0.7	0.74	0.40	0.57	1.25	0.69	0.54	0.46	0.52	0.34	0.12	0.53	0.93	0.61	0.46	0.39	0.78
C13:0	0.08	0.05	–	0.10	–	–	0.11	0.09	–	–	–	–	–	–	–	–	–	–	0.14	0.09	0.14	0.15	0.14	0.06	–	0.12	0.09	–	–	0.09	0.14
C14:0	1.24	0.91	0.88	0.91	0.55	1.35	0.68	0.70	0.32	0.49	1.15	–	0.85	0.91	0.91	2.40	0.66	0.72	1.99	1.69	1.21	1.27	0.99	1.00	0.57	1.65	1.00	–	0.86	0.86	1.32
C15:0	0.32	0.35	0.28	0.42	0.22	0.41	0.18	0.25	–	–	0.48	–	0.27	–	–	0.34	0.46	–	0.57	0.42	0.47	0.49	0.48	0.32	0.12	0.55	0.42	–	0.51	0.40	0.59
C16:1	0.22	0.26	–	0.28	0.56	0.24	0.28	0.26	0.13	–	0.71	–	–	–	–	–	0.14	–	0.23	–	–	0.30	0.06	0.16	0.14	0.54	0.31	–	–	0.30	0.24
C16:0	8.56	6.47	6.00	16.57	5.99	12.67	2.15	4.92	1.65	12.06	14.58	3.77	22.99	5.74	17.8	7.63	14.12	8.20	8.86	8.80	12.36	11.13	11.99	17.60	1.33	7.62	5.90	2.88	9.56	13.34	19.40
C17:0	0.18	0.40	–	0.58	–	–	–	–	–	–	–	–	–	–	–	0.80	–	–	0.18	–	–	0.45	0.35	0.76	–	–	–	–	–	–	0.27
C18:2	–	–	–	–	–	–	–	–	–	–	–	–	–	–	–	0.63	–	–	–	–	–	–	–	–	–	–	–	–	–	–	–
C18:1	0.74	0.99	0.63	1.21	0.85	0.99	0.76	0.69	0.55	4.54	3.33	1.82	1.95	2.47	5.41	1.12	1.74	2.95	0.75	0.73	0.43	2.37	1.84	5.69	0.68	1.70	2.06	0.96	0.61	3.59	1.76
C18:0	7.31	5.41	8.14	17.61	7.39	14.40	1.53	4.11	1.13	5.97	11.58	0.54	22.44	2.79	9.18	10.18	14.41	7.82	8.76	8.3	19.95	12.79	12.35	16.50	0.98	6.39	5.60	2.54	7.05	8.77	10.82
C20:0	–	0.61	–	0.68	–	–	–	–	–	–	–	–	–	–	–	1.65	–	–	0.58	0.31	0.57	–	0.32	0.52	–	–	–	–	–	0.41	0.66
C22:0	–	–	0.34	–	–	0.35	–	–	–	–	–	–	–	–	–	0.66	0.60	–	1.55	1.56	1.37	0.82	0.98	0.84	–	1.10	–	–	0.82	1.00	1.85

Eighteen acids, primarily saturated, were determined in the samples. C16:1 and C18:1 represented the group of unsaturated acids. C18:2 was determined in sample 113 from Domasław. C6:0, C8:0, C9:0, C10:0, C12:0, C16:0, C18:1 and C18:0 acids were present in all samples. Long-chain acids—C20:0 and/or C22:0—were detected in most samples from Milejowice, and in six from Domasław. C11:0 acid was identified in vessels 42 and 43 from votive pits in Milejowice. This acid was also rare at the cemetery, recorded in urns, *rhyta*, a censer, and offering vessels. Statistical analyses showed that settlement and burial vessels differentiate most acids C13:0, C15:0, C22:0 and the C22:0/(C16:0 + C20:0) ratio.

Any conclusions about the sources of the examined organic residues based solely on the presence and content of individual acids would be unreliable. Most acids are present in both plant and animal resources. Moreover, the residue composition can change over time, which should always be considered in archaeological samples. Consequently, researchers are looking for another way to interpret the results of chromatographic analyses. One of the methods is based on studying the proportions of selected fatty acids, as it appears that acid proportions can remain unchanged over time. The conclusions presented in this paper were based on the work of Eerkens^20^. Based on his proposed proportions, the most likely sources of the studied residues were designated. The calculated proportions of selected acids are shown in Table 3.

**Table 3:** Calculated proportions of selected fatty acids in the studied samples.

Sample number	Fatty acid proportion				
	(C15:0 + C17:0)/C18:0		C16:1/C18:1	C16:0/C18:0	C12:0/C14:0
Domasław cemetery					
3	0.07	0.30		1.17	0.40
4	0.14	0.26		1.20	0.51
10	0.03	–		0.74	0.58
12	0.06	0.23		0.94	0.41
90	0.03	0.66		0.81	0.84
91	0.03	0.24		0.88	0.64
93	0.12	0.37		1.41	0.84
94	0.06	0.38		1.20	0.87
95	–	0.24		1.46	1.06
101	–	–		2.02	1.18
104	0.04	0.21		1.26	0.59
105	–	–		6.98	–
106	0.01	–		1.02	0.48
108	–	–		2.06	0.90
111	–	–		1.94	0.77
113	0.11	–		0.75	0.31
Milejowice settlement					
39	0.03	0.08		0.98	0.61
40	–	–		1.05	0.79
42	0.09	0.31		1.01	0.63
43	0.05	–		1.06	0.41
46	0.02	–		0.62	0.45
47	0.07	0.13		0.87	0.36
49	0.07	0.03		0.97	0.53
53	0.07	0.03		1.07	0.34
55	0.12	0.21		1.36	0.21
58	0.09	0.32		1.19	0.32
61	0.08	0.15		1.05	0.93
70	–	–		1.13	–
72	0.07	–		1.36	0.53
79	0.05	0.08		1.52	0.45
86	0.08	0.14		1.79	0.59

The acid proportions suggest that most examined samples have a mixed origin. The residue probably came mainly from seeds, nuts, and berries in combination with fat from land mammals (samples 39, 42, 47, 55, 58, 61, 79, 86 from Milejowice and 3, 4, 12, 91, 95, 104, 105 from Domasław). The proportions in grave samples 90, 93, 94 suggest seeds, nuts and land mammal fat. Vessels 40, 43, 46, 49, 53, 72 from the settlement and 10, 101, 106, 108, 111, 113 from the cemetery were of plant origin (nuts and seeds, berries), 70 from Milejowice – originated from seeds and nuts. The C16:0:C18:0 ratio has long been used to distinguish different plant oil sources, with a high (> 3) ratio considered characteristic of poppy seed oil^21^. At vase 105, the ratio was 6.98. A higher C18:1 ratio was detected in *rhyta*, vases and pots from the cemetery, including most vessels from grave No. 7429 with vases 101 and 104. Scholars suggest that dicarboxylic acid compounds are more likely to be found in plant oils^22^. The presence of dicarboxylic acids and oleic acid (C18:1) increases the possibility that the samples contained acid-rich plant oil or derivative mixtures.

Gas chromatography combined with mass spectrometry made it possible to determine organic compounds from different groups. Based on a literature review, it was possible to select compounds that can be considered archaeological biomarkers. Biomarkers are associated with one specific material or substance, regardless of its origin—plant, animal or mixed. The detected fat is not necessarily an oil but may have been an ingredient of herbs, vegetables or grains.

Methyl dehydroabietate, a dehydroabietic acid derivative, was detected in almost all samples from Milejowice (except 42, 43, 55) and seven from Domasław (3, 4, 12, 90, 91, 93, 94). This acid is classified as a resinous acid and is considered an indicator of resin or its products^23–25^. Resins have hydrophobic properties and can be used to seal unglazed vessels^26^, but they have also been intentionally added to alcoholic beverages to preserve, enhance, and change their flavour^27–30^. Adding resin to wine to protect it against disease, for medicinal purposes and to cover up off-tastes and off-aromas was a popular and widespread practice throughout the ancient world^31^. Cedrol—sesquiterpene alcohol, found in the essential oil of coniferous trees, was present in settlement vase 55.

Glycerol, a product of lipid degradation, was also observed in most samples from Milejowice (except 55, 79, 86) and in more than half of the samples from Domasław (3, 90, 91, 93–95, 101, 104, 106). Vanillin, a phenolic compound, and vanillic acid (found in elderberry juice, blueberries, strawberries, and alcoholic beverages^32^) were detected in nine vessels from Milejowice (39, 40, 46, 53, 55, 58, 61, 70, 72) and in all samples from Domasław except 101, 106, 108, 111, 113. Vanillin may originate from tree resin or pine wood/dust, which is documented in the same samples by the presence of methyl dehydroabietate. Acetowanillone, identified in settlement vase 70, a structural analogue of vanillin, is found mainly in wines aged in oak barrels.

Lactic acid, determined in nine vessels from the settlement (39, 40, 42, 46, 47, 49, 58, 61, 70) and eight from the cemetery (4, 10, 90, 91, 93, 94, 95, 104), is one of the essential products of bacterial fermentation, which occurs, e.g., during vegetable pickling or milk fermentation. However, malic acid (a beer and wine ingredient) can also be converted by bacteria into lactic acid, which gives the beer a sour taste^33,34^.

Azelaic acid was identified in nine vessels from the settlement (39, 40, 42, 49, 53, 55, 58, 61, 86) and seven from the cemetery (4, 10, 12, 93, 94, 101, 105); oxalic acid in four from Milejowice (40, 46, 55, 58) and three from Domasław (12, 91, 95); suberic acid in five from the settlement (42, 53, 55, 58, 61) and two from the cemetery (4 and 12). Their presence may suggest that the studied residues originated from grain products, including wheat, rye, or barley^35,36^.

Suberic acid is produced also during the oxidation of castor oil and is used to produce resins. Acetic acid, observed in vase 4, may indicate the contact with fermented food^34^. It is formed from ethanol under the action of aerobic acetic bacteria (vinegar production), as well as from acetaldehyde, which, as a component of beer, gives it its characteristic flavour and aroma^33–35^. Fumaric acid, confirmed during beer fermentation and wort production^34^, was present exclusively in vase 94 from Domasław. Only settlement vessel 53 produced traces of caprolactone, a fragrance component found in flowers, some fruits and vegetables. Δ-Caprolactone is found in heated milk fat^37^.

Stigmastanol, present in four samples from Domasław (4, 12, 93, 95) and seven from Milejowice (40, 43, 55, 58, 70, 79, 86), also indicated their plant origin. This sterol occurs in vegetable fats or oils of many plants: beans, rapeseeds and herbs. Another noteworthy example is benzoic acid, determined in half of the samples from the necropolis (90, 91, 93, 94, 101, 104, 106, 113) and only one from the settlement (53). Its presence may also indicate that the examined residues originated from plants, as it is a component of plant tissues, especially fruits and vegetables^38^. This acid is detected, for example, in cherry bark, raspberries, and honey. Benzoic acid may also be a degradation product of anthocyanins by ketones in wine^39^.

Of particular importance may be the tiglic and croton acids present exclusively in vase 12 from the Domasław cemetery and 13 containers from this site, such as *rhyta*, a kernos, censers, a disc-plate, an urn, and offering vessels. Tiglic acid is a compound with a spicy smell, it occurs, among other things, in croton oil obtained from the seeds of laxative croton, a plant from the *Euphorbiaceae* family, used for medicinal purposes.

Borneol, which appeared in two vases from the cemetery (10, 90) and four from the settlement (39, 42, 55, 79), is a compound from the terpene group with a camphor-like fragrance. It is a component of many essential oils, pine resin, and herbs, e.g., tansy, savoury, sage, and plants from the wormwood family. Like croton oil, it has medicinal and toxic effects and may cause eye and skin irritation and vomiting. Borneol was found mainly in the most distinctive vessels from the cemetery, such as an urn, a *rhyton* and offering vessels, At the Milejowice settlement, it occurred in dippers, a plate-disc and bowls. Phenoxyethanol was detected only in funerary vases 90, 91, 93, 94 and 95, two miniature *rhyta* and an urn. It can be found naturally in some plants, such as onions and chicory, and helps control and prevent the growth of bacteria, yeasts and moulds. Inulin, an extract from chicory root, has been used as a sweetener. Some beer brewers use roasted chicory to add flavor to stouts, or to augment the hops. Roots contain essential oils similar to those found in plants in the related genus *Tanacetum*. Carvacrol was detected only in three vases from Milejowice (55, 58, 61). There are many sources of carvacrol, including various kinds of thyme and marjoram.

Adipic acid from three funerary samples (90, 91, 101) occurs naturally in beets and sugarcane. Gramine (donaxin), found in funerary vase 101, is a naturally occurring indole alkaloid found in several plant species, such as silver maple and *Hordeum*, a genus of grass that includes barley^40^. 9,12-Dihydroxyoctadecanoic acid (Z,Z), present in a funerary vase with a funnel-shaped rim and conical neck (101) and in all vases with cone-shaped necks (105, 106, 108), is a linoleic acid derivative and a component of most vegetable oils and animal fats, found in many foods, e.g., legumes, cloves and nuts. It is produced through the hydration of ricinoleic acid, which is found in large amounts in castor oil and has historically been reported to be a significant resource with various uses in ancient times.

3,4-dihydroxybutyric acid found in vase 72 from Milejowice, is believed to be formed via the degradation (cooking) of di- and polysaccharides, including lactose. Farnesol, an alcohol from the terpene group obtained from linden flower oils, is used mainly as the lily-of-the-valley fragrance and is a component of many essential oils, including lily of the valley, linden and acacia. It was found in painted vases 47 and 49 from the settlement.

The contents of funerary vessel 113 proved very distinctive, suggesting the presence of plants and honey. The detected spirost-8-en-11-one, 3-hydroxy- is a component of sugar plants. The sample also contained 7,8-epoxylanostan-11-ol, 3-acetoxy, which is a cholesterol-based alcohol with anti-inflammatory and antibacterial properties that occurs in many plants. Furthermore, a propolis ingredient, 17-pentatriacontene, 1-heptatriacotanol, was identified. Additionally, 1-heptatriacotanol, an alcohol with antioxidant, anticancer, and anti-inflammatory properties, was confirmed. It occurs in various waxes, e.g., beeswax or plant waxes (jojoba oil). Last, the studies detected 1-monolinoleoylglycerol—an ether, glyceride consisting of one chain of fatty acid (linoleic acid) and glycerol. It occurs, among others, in plants of the *Lamiaceae* family, which are used as spices, medicines and teas.

Valproic or valeric acid was present in vases 90 and 91 from the cemetery and vessels 42 and 43 from settlement votive pits. Valproic acid is a saturated monocarboxylic acid and a valeric acid derivative. It occurs naturally in some foods, such as berries. It has sedative and anticonvulsant properties.

The presence of dibutyl phthalate, a phthalic acid present in six vessels from Milejowice and nine from Domasław, may indicate the thermal processing of products in vessels^41^ or may result from sample contamination.

### Acid data analysis and classification

**Figure 2:**
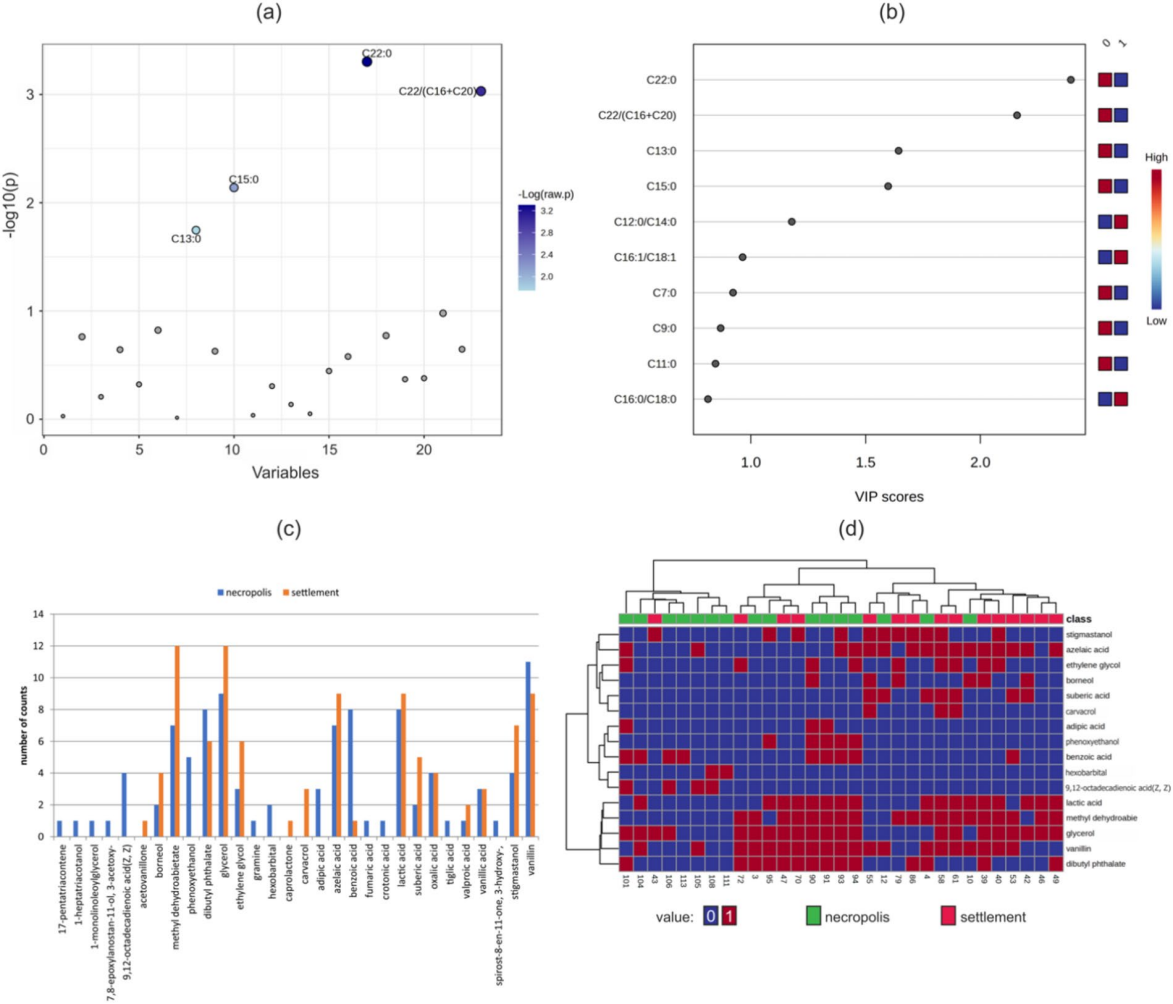
Important features selected for sample classification: (**a**) features of subset 1 selected by *t*-tests with a significance threshold of 0.05. Blue circles represent features above the threshold (p); values are transformed by –log10; (**b**) Features of subset 2 selected by the VIP value (score above 1). Characteristics of the biomarker distribution in vase samples, including a grouped histogram of biomarker occurrences in samples from the cemetery and settlement (**c**) and a clustered heatmap of biomarkers for which the minimum absolute difference between the two groups of vases in histogram was at least 2. Additionally, the frequently occurring lactic acid was considered (**d**).

In this study, based on acid-related data, we applied the FCM algorithm to cluster samples of ceramic vases from the settlement in Milejowice and the cemetery in Domasław. Features important for the data analysis were selected by *t* tests with a threshold of 0.05 and VIP values were obtained with PLS regression (Fig. 2).

The results were two subsets of variables used independently in the analysis. Subset 1 includes acid-related variables such as C13:0, C15:0, C22:0 and C22:0/(C16:0 + C20:0) (Fig. 2a,b). The set of variables with the highest VIP value (> 1) included the same components, except for the additional C12:0/C14:0 acid proportion. The test values are listed in Table 4.

**Table 4:** Values of the *t*-statistics of the Student’s *t*-test for raw data regarding acids and their proportions in two groups of vases from different locations (independent groups).

Variable	*t* stat	*p* value	*VIP* value
C22:0	4.4996	0.00038	2.3949
C22:0/(C16:0 + C20:0)	3.8167	0.00131	2.1602
C13:0	2.6602	0.01365	1.6439
C15:0	2.5888	0.01518	1.5983
C12:0/C14:0		n.s.	1.1784

*n.s.* not significant.

**Figure 3:**
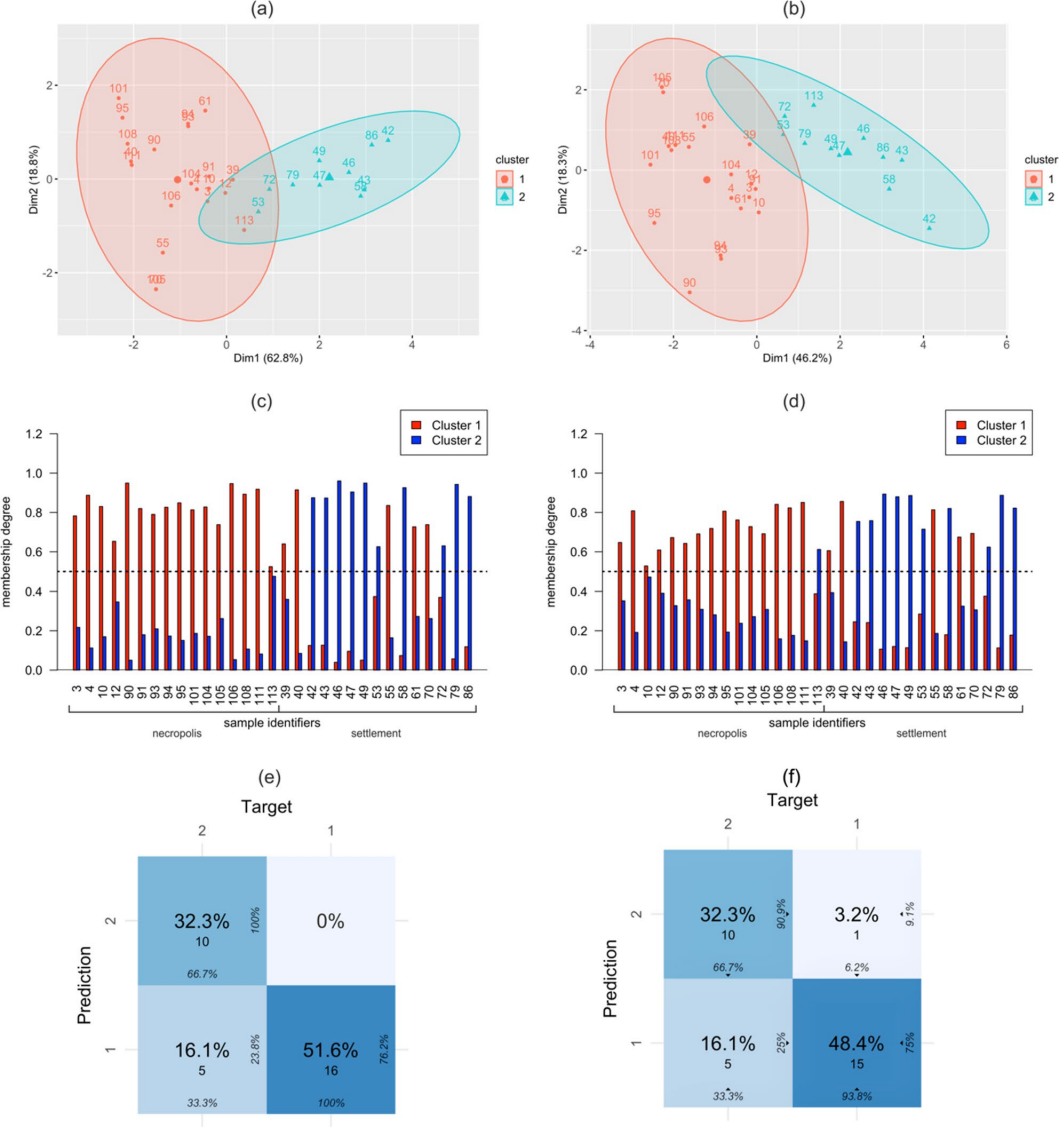
Illustration of the clusters of archaeological samples from the cemetery and the settlement in the plane of the PCA main components for the selected feature subset 1 (**a**) and subset 2 (**b**). Each ellipse is a 95% confidence ellipse for a 2D normally distributed data set. The probability of samples belonging to clusters 1 or 2 based on the selected acid data is also presented for feature subset 1 (**c**) and subset 2 (**d**). Confusion matrices for feature subset 1 (**e**) and subset 2 (**f**).

Using the fuzzy *C*-means method, the dataset was grouped into two clusters, where each data point belonged to each cluster to some extent. Clustering results are shown in Fig. 3a and Fig. 3b. The obtained results show that the clusters largely correspond to the sample origin. The probability plot for feature subset 1 (Fig. 3c) shows that cluster 1 contains samples from the necropolis and five samples from the settlement, while cluster 2 contains only samples from the settlement. Adding acid proportion C12:0/C14:0, another considered feature slightly reduced the probability that the samples belonged to the selected clusters (Fig. 3d) but did not significantly disturb their previous distribution. With the data in Fig. 3c,d, adopting more flexible grouping rules, e.g., more restrictive than a classification cut-off at a probability of 0.5 was possible. The requirement of a higher probability of class membership leaves some samples out as unclassified due to uncertainty.

Figure 3e,f represent the confusion matrices obtained for the two feature subsets. They confirm the obtained probability results and quantify the quality of the archaeological sample classification. The ratio of the sum of diagonal elements to the sum of all matrix elements represents the classification accuracy obtained from clustering. For both matrices, it is 0.84 and 0.81, respectively. It means that features selected by the Student’s *t*-test provide a better separation of clusters than features selected by the PLS-DA method and, simultaneously, ensure better compliance with their actual division.

Table 5 includes three different indicators of fuzzy clustering effectiveness explained in Eqs. (1)–(3).

**Table 5:** Evaluation of the clustering results for selected acid data.

Validation index	Subset 1	Subset 2
Accuracy	0.8387	0.8387
Fuzzy silhouette index (SI)	0.7644	0.6745
Dunn partition coefficient (DC)	0.7910	0.7320
Partition entropy index (PEI)	0.3471	0.4274

Dunn’s partition coefficient (DC) reaches 0.79, which suggests that the clustering is not very fuzzy, and the crisp sample grouping methods can also be applied. Partition entropy index PEI = 0.35 in the range [0,1] informs that the uncertainty within the proposed partition is relatively low, which confirms the observations about the DC. The Fuzzy Silhouette Index value of 0.76 leads to the same conclusions.

### Biomarker data clustering

The vessels yielded different biomarkers, most notably benzoic acid, vanillin, and phenoxyethanol. The analysis of histogram and clustering results revealed that some biomarkers were more common in settlement samples (Fig. 2c,d). These included methyl dehydroabietate, glycerol, azelaic, suberic, adipic acids, and stigmastanol. Vanillin, and benzoic acid were most frequent in cemetery samples. Grave vases had a higher plant oil content.

Vases were most differentiated by the benzoic acid, which were detected in 62.5% of funerary vessels and only 6.5% of settlement vessels. Lactic acid was present in equal proportions in settlement vases and funerary vases with funnel-shaped rims. However, there was no lactic acid or dibutyl phthalate in grave vases with distinguished conical necks. In contrast, 9,12-octadecadienoic acid occurred in all vases with conical necks, one vessel with a funnel-shaped rim, and in none of the settlement containers.

The cluster analysis of the identified biomarkers showed that vessels from the settlement were more similar to one another than were vases from the cemetery. Sample 113 differs from the others and is closer to the pattern recorded in settlement vases.

## Discussion

The organic residue analysis demonstrated that most of the vases from the cemetery and settlement were of a mixed nature, originating from seeds and nuts and berries, and approximately 40% had a plant character. The vases might have come into contact with food based on cereals, herbs, honey, fruit, animal fat (meat or milk), oily plant products and fruits, bee products, and resins. Due to their design, vase-shaped vessels were preferred for storing drinks. Cereal cultivation was confirmed by botanical analyses of samples from Domasław, which showed the presence of burnt wheat and millet grains in vessels and graves (studies by A. Sady-Bugajska). We can assume that vessels contained particularly fermented beverages, most likely *ale gruit* of wheat, barley and/or rye, as indicated by markers of these cereal species. Chemical evidence of millet (miliacin) combined with possible fermentation markers (bacteriohopanoids) in drinking vessels from Mont Lassois seems to confirm the production of millet beers^42^.

Plant ingredients, e.g., stigmastanol, cedrol, herbal ingredients, such as carvacrol and borneol, and bactericidal or medicinal ingredients (valproic acid) could be added to beverages as flavourings, to extend their storage time (preservatives), but also to enhance their effect. The addition of herb, resin and honey imparted different drink properties and increased variety. Phenolic compounds, like vanillin, may come from malt (sprouted and dried cereal grains) and affect the flavour and clarity of beverages. Simple phenols may also be derivatives of benzoic acid present in the samples^43^. The presence of tiglic and croton acid may indicate that the vessels contained oils added for medicinal purposes, flavouring and symbolic reasons, which is suggested by their presence only in vessels associated with offerings.

We may suspect mead in vessel 113. Relatively few bee products were found in the vases, which is surprising considering the very sour or tart taste of the ancient beer (unless we consider the presence of benzoic acid in the funerary vessels as a possible propolis residue). Honey and beeswax frequently occur in archaeological ceramic containers—the former as a sweetener and the latter as a vessel sealant. Honey is documented mainly by pollen analyses, among others, in bronze craters from princely graves in Heuneburg-Hohmichele, Heuneburg-Speckau, Hochdorf, Glauberg and Niedererlbach^44–49^, or recognised as a honeycomb in Lavau^50,51^.

Some fruit components (benzoic, valproic, levulinic acids, vanillin) may come from fruit wine and sugars from tree saps, e.g., birch and maple, which were undoubtedly consumed both fresh and fermented. It is possible that alcoholic barley milk beverages, made by mixing malt extract with milk were also consumed. Alcohol made from sap or fruits was archaeologically attested^42^. There is also evidence for combining beers, wines, and meads to make mixed beverages called grogs. Nordic peoples preferred a hybrid beverage, in which many ingredients were fermented together, including locally available honey, fruits, cereals, and sometimes imported grape wine^52^. Birch tree resin, juniper, bog myrtle, yarrow, bog cranberry, and lingonberry were also used as additives.

Archaeological evidence shows that feasting was significant in the Hallstatt period and gave political power^53–56^. Drinking together and providing alcohol forged social prestige and demonstrated power and wealth. Almost all elite graves from this period contained vessels for distributing alcohol. Similar vessels from settlements emphasised the role that alcohol played during feasts, festivals, meetings and celebrations. The most spectacular pieces of libation pottery are segmented vases with funnel-shaped rims and conical necks, which appeared at the onset of the Iron Age due to the influence of Hallstatt. Through these “Hallstatt” patterns, vases also highlighted their owners’ status. The Domasław cemetery and the Milejowice settlement yielded many references to feast and libation rituals. In Milejowice, the massive, decorated vases 42 and 46, containing other vessels, animal bones, and bronzes, undoubtedly had a votive character, probably similar to containers from the well (39, 40). Potentially, they had initially served for storage or fermentation but were later used in ritual activities as food or drink sacrifice accessories. Other vessels are not that distinctive. Despite their inconsistent distribution patterns, scholars indicate that graphite-coated and painted vases with funnel-shaped rims and conical necks were more “prestigious” than daily household vessels^5^. Containers with fermenting liquids must be well-sealed with wax or resins and buried in the ground, like some larger storage vessels discovered in Milejowice, e.g., with identified tar. Smaller ones might, in turn, have been used to distribute (perhaps diluted) alcohol.

The vessel sets discovered in Domasław were undoubtedly used for storing, serving and consuming beverages. It seems that vessels with funnel-shaped rims, most often deposited pairwise, were used by an increasing number of people during funerary ceremonies. Specimens from the early Hallstatt period were smaller, but they became the largest funerary vessels in graves over time. These vessels were surrounded by small cups and bowls, often placed inside them in substantial numbers. Sometimes, plate-shaped bowls were placed as lids on top of the vases. Two of the examined vases contained small bronze vessels which, as shown in the depictions of the late Hallstatt period Situla Art in the Southeast Alps^57^, were used to serve alcohol. Some depictions also show dry products measured in large vases, such as wheat grains, semolina, herbs, raisins or cheese. Vases with cone-shaped necks probably played a different role and were repeatedly placed next to crater-shaped vases, often covered, which may explain differences in the share of, among others, benzoic acid. A comparison of vases from the same graves revealed that they contained different products.

We might assume that decorated vases, both at the settlement and the cemetery, served as beverage containers. Undoubtedly, the products used for daily and funerary activities were diverse, and some might have been associated with taboos. This may explain the differences between the vessels found in these two contexts. The study demonstrated that some compounds were present only in vessels from one context.

The consumed *ale* was flavoured with a selected mixture of herbs (*gruit*). It is possible that each family/community had its original herbal mixture or recipe passed down from generation to generation, or the composition was subject to various instructions and prohibitions. Different herbal mixtures based on nearby plants were used depending on the occasion for which the beer was prepared. Like hops today, *gruit* was supposed to preserve the drink and give it the right taste, aroma, and desired properties. The principal bittering agents in the early medieval European beers were bog myrtle, yarrow, meadowsweet, and other herbs^43^. For example, native rosemary, mint, and thyme were added to a fermented emmer wheat and barley beverage at Genó, near Barcelona in Spain^58^. Mugwort was a hypothetic additive (alongside carrot) to a dark, sour barley beer^59^ at the settlement of Hochdorf.

Some plants with symbolic, flavouring, intoxicating, medicinal or poisonous properties could only be used in funerary and offerings contexts (e.g. croton oil). Presumably, flour/groats/barley, milk/cheese, wine/beer, and even blood, mentioned in the ancient sources as obligatory for libations and sacrifices were used, which could explain the combination of animal and plant fats in the vessels. Animal fats may also have come from the leather bottles used to transport liquids. The multitude of uses of resins and bee products, such as sealing, polishing, preserving, sweetening, flavouring., does not allow us to assess for what purpose they were used in Domasław and Milejowice. The resins were more common in settlement vessels, which may have been caused by the need to seal containers that are used repeatedly. Some aromas, oils, and spices could be imported, similar to other prestigious products.

## Conclusions

Analysis of vases from the Domasław cemetery and Milejowice settlement reveals that they were used to store a variety of beverages, including beer made from wheat, barley, and rye. These vessels contained a diverse range of ingredients, such as fruits, herbs, plant oils, animal fats, honey, and resins. Their content varied in funerary and settlement contexts due to the use of certain plants as well as specific ingredients that imparted the desired properties.

Feasting was crucial during the Hallstatt period, demonstrating social status and power, as evidenced by the presence of drinking sets in elite graves. Richly decorated vases found in the cemetery and in the offering settlement context, suggest their important role in ceremonies. The study highlights the complexity of food and drink preparation, providing insights into the eating habits and rituals of the Hallstatt communities. Consequently, it is a foundation for further exploration of the nature of vessels and the assessment of different consumption practices within the community.

The statistical analysis proved effective in capturing the differences between chemical profiles of vessels deposited in two distinctive contexts, as well as the use of various ingredients in graves, deposits and settlement features. Archaeological data, due to their complexity and often incompleteness, are among the most difficult to analyze and interpret. Hence, this article utilizes statistical analyzes and machine learning methods as a tool of facilitating further interpretation of chemical data. The clustering method used showed the statistical differences in chemical compositions, which may be ambiguous for single samples, given the possibility of multi-faceted use of individual types of prehistoric vessels.

## Data Availability

The datasets used and/or analyzed during the study are reported in the article.
